# The Role of miRNA in Tumor Immune Escape and miRNA-Based Therapeutic Strategies

**DOI:** 10.3389/fimmu.2021.807895

**Published:** 2022-01-18

**Authors:** Zhengjia Zhang, Qingcai Huang, Liuchunyang Yu, Dongjie Zhu, Yang Li, Zeyu Xue, Zhenglai Hua, Xinyi Luo, Zhiqian Song, Cheng Lu, Ting Zhao, Yuanyan Liu

**Affiliations:** ^1^ School of Chinese Materia Medica, Beijing University of Chinese Medicine, Beijing, China; ^2^ Institute of Basic Theory, China Academy of Chinese Medical Sciences, Beijing, China; ^3^ Institute of Basic Research in Clinical Medicine, China Academy of Chinese Medical Sciences, Beijing, China

**Keywords:** tumor immune escape, miRNA-based tumor therapy, innate immune response, specific immune response, tumor cell apoptosis, exosomes

## Abstract

Tumor immune escape is a critical step in the malignant progression of tumors and one of the major barriers to immunotherapy, making immunotherapy the most promising therapeutic approach against tumors today. Tumor cells evade immune surveillance by altering the structure of their own, or by causing abnormal gene and protein expression, allowing for unrestricted development and invasion. These genetic or epigenetic changes have been linked to microRNAs (miRNAs), which are important determinants of post-transcriptional regulation. Tumor cells perform tumor immune escape by abnormally expressing related miRNAs, which reduce the killing effect of immune cells, disrupt the immune response, and disrupt apoptotic pathways. Consequently, there is a strong trend toward thoroughly investigating the role of miRNAs in tumor immune escape and utilizing them in tumor treatment. However, because of the properties of miRNAs, there is an urgent need for a safe, targeted and easily crossed biofilm vehicle to protect and deliver them *in vivo*, and exosomes, with their excellent biological properties, have successfully beaten traditional vehicles to provide strong support for miRNA therapy. This review summarizes the multiple roles of miRNAs in tumor immune escape and discusses their potential applications as an anti-tumor therapy. Also, this work proposes exosomes as a new opportunity for miRNA therapy, to provide novel ideas for the development of more effective tumor-fighting therapeutic approaches based on miRNAs.

## 1 Introduction

Cancer has become the leading cause of death, with a high incidence and low cure rate, and a major impediment to extending life expectancy due to a lack of specific therapy worldwide (1). Given the high degree of deregulation of the immune system during tumor genesis and progression, restoring immune system balance is a potentially effective and specific anti-tumor therapy. Immunotherapy, which represents a paradigm shift in oncology treatment, aims to overcome the immune suppression caused by the tumor microenvironment and restore the immune system so that it can target and kill tumor cells *via* immune response while also promoting tumor cell apoptosis (2). However, under the selective pressure of immune surveillance, tumor cells undergo continuous remodeling at the genetic and epigenetic levels and develop a series of escape mechanisms, for example, influencing the immune response process or resisting apoptosis, to escape from immune surveillance; this results in the drawbacks and off-target of immunotherapy with low clinical response and drugs susceptibility (3, 4). Immune escape is not only a major cause of immunotherapy failure, but it is also a necessary process for tumors to begin their malignant progression (5). Considering that this process is characterized by genomic instability (6), the escape mechanisms by tumor cells, including immune response disruption, resistance to apoptosis, or reduced immune recognition, may cause structural changes or deregulated expression of genes/proteins in both tumor cells and immune cells (7). Along with these genetic and epigenetic changes, tumor immune escape is frequently associated with small non-coding genome elements, known as miRNA.

**Figure 1:**
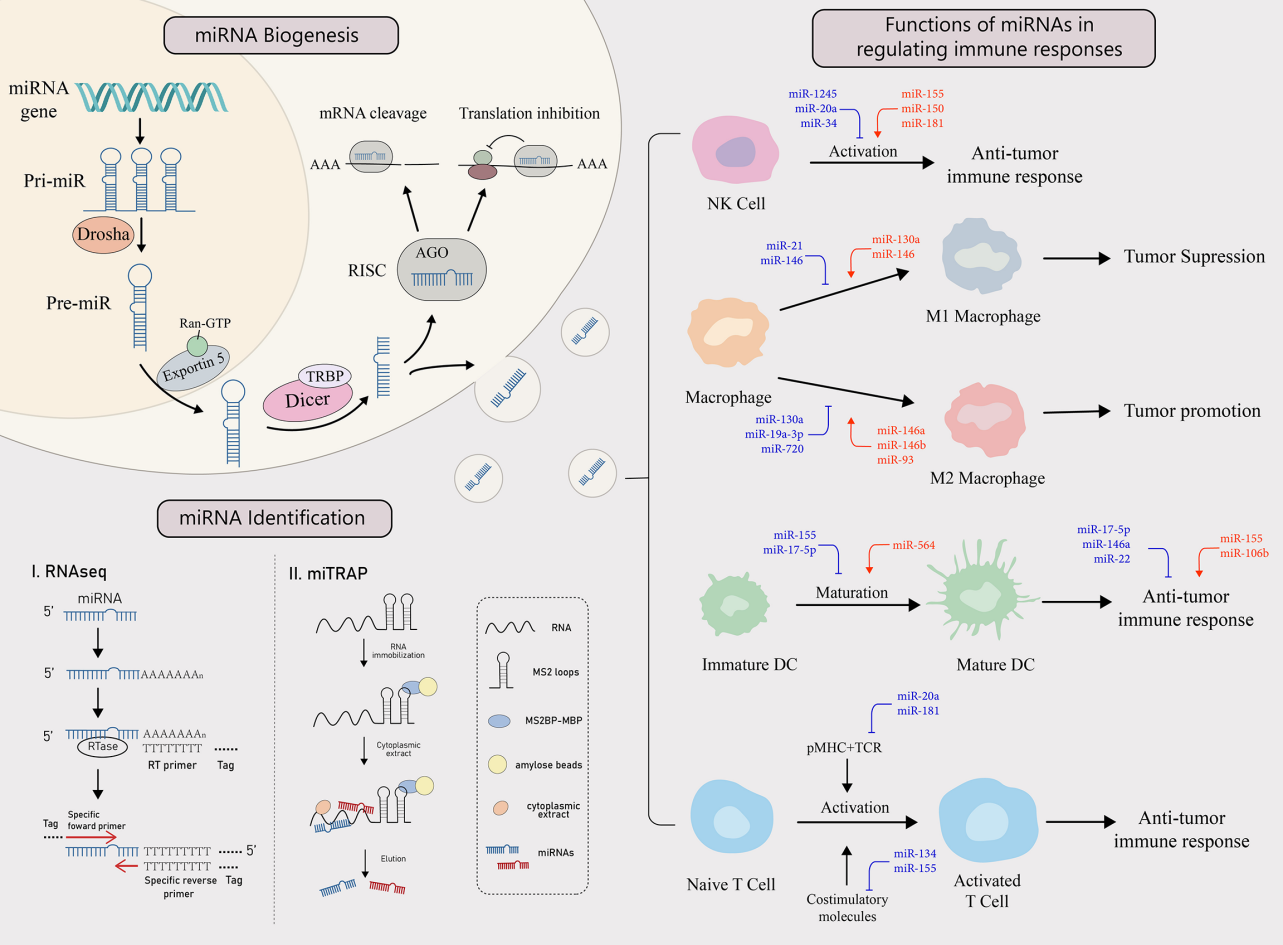
miRNA biogenesis, identification and functions in regulating the immune response. miRNA biogenesis: The biogenesis of miRNA begins when miRNA gene is transcribed into primary miRNA (pri-miRNA). It is then cleaved by Drosha, a RNase, to form pre-miRNA, which enters the cytoplasm from the nucleus with the help of exportin 5 and the RAN-GTP complex. There, after Dicer and TAR RNA binding protein (TRBP) bind to the pre-miRNA, one of the strands is incorporated into the RNA-induced silencing complex (RISC) to form a mature miRNA that performs its functions. miRNAs target mRNAs with complementary sites in the 3’UTR and cause either translation inhibition or mRNA cleavage. miRNA identification: There are two main ways to identify miRNAs, RNAseq and miTRAP. miTRAP analysis is a technique for sequencing miRNAs using high-throughput sequencing technology to reflect their expression levels. [miTRAP analysis is a technique that captures miRNAs by *in vitro* affinity purification of posterior RNA. miRNAs in the cytoplasm can also be delivered out of donor cells *via* exosomes and become a tool for intercellular communication. Functions of miRNAs in regulating immune responses: Tumors can influence the activation, differentiation, maturation, and function of various types of immune cells through the secretion of specific miRNAs, thereby regulating immune response. The red miRNAs represent a promoting role, while the blue miRNAs play an inhibiting role.

miRNAs are small non-coding single-stranded RNAs of approximately 22 nucleotides long, silencing targeted mRNAs at the posttranscriptional or translational level by binding to the 3’ untranslated region (3’UTR) of the mRNA to inhibit the translation process or directly cut it (8, 9). Tumor cells and the tumor microenvironment (TME) are constantly reshaping themselves directly or indirectly through an aberrant expression of specific miRNAs, that have, tumor-promoting or tumor-suppressing effects. In TME, miRNAs are thought to be an important molecular mechanism for mutual interference between tumor cells and immune cells. When the function of immune cells against tumors is disrupted by specifically secreted miRNAs, the immune responsive process fails to clear tumor cells (**Figure 1**). Meanwhile, tumor cells may actively reprogram themselves to escape apoptosis by expressing aberrant miRNAs, allowing tumor cells to proliferate and deteriorate uncontrollably (6).

The potential role of miRNAs as tumor suppressors or oncogenes (oncomiRs) to participate in tumor progression as well as their heterogeneous functions across tumor types, make them an appealing therapeutic tool for targeting immune escape (10). Hence, therapeutics using miRNAs alone fall into two categories: (i) restoring tumor suppressor miRNA levels, including miRNA mimics and small molecule drugs; (ii) blocking oncomiR functions, such as antagomiRs, locked nucleic acids (LNA), and miRNA sponges (11). Based on the foregoing, miRNAs emerge as a widely used therapeutic candidate with a strong potential to become adjuvants for several major immunotherapies currently in use, synergistically improving immunotherapeutic efficacy and reducing off-target effects (12). However, because naked miRNAs have a short half-life *in vivo* and are easily degraded, it is urgent to seek a safe, effective, and targeted vehicle to protect and deliver them to their intended sites. Exosomes have emerged as the most promising vehicles for miRNAs in recent years, owing to their high permeability across biological barriers, the long half-life, and natural ability to function as shuttle carriers for cargo transfer under both physiological and pathological conditions (13). Exosomes are typically recognized by the recipient cell *via* antigen-antibody or receptor-ligand interactions, triggering a signaling cascade that activates the endocytic pathway, allowing the exosomes to enter the recipient cell and release contains *via* phagocytosis or pinocytosis (14).

In this paper, we first review the role of miRNAs in immune escape on three aspects: innate immune response, specific immune response, and tumor cell apoptosis. After that, we summarize miRNA-based applications, either alone or in combination with other immunotherapies, and highlight exosomes as a promising carrier of miRNAs. This review aims to suggest a critical role of miRNAs in tumor immune escape and elucidate the precise mechanisms regulated by miRNAs, required for a deeper understanding of immune escape, as well as provide opportunities for their development as therapeutic targets, thereby improving the efficacy of specific immunotherapy on tumors.

## 2 miRNA-Regulated Innate Immune Response Through Innate Immune Cells

The innate immune response is the first stage of immune system activation, and it is characterized by an immediate response associated with non-specific recognition and killing of tumor cells. Alterations in miRNA expression profiles in tumors may result in altered or reversed functions and phenotypes of natural killer cells (NK cells), macrophages and dendritic cells (DCs), all of which are major components of the innate immune response. With impaired or altered function of immune cells, tumor cells may escape the surveillance of innate immunity. **Table 1** summarizes the expression levels, targets, functions, and clinical relevance of some miRNAs in different immune cell populations (**Table 1**).

**Table 1:** Summary of miRNAs expression levels, targets, functions, and clinical relevance of different immune cell populations.

Immune cell Populations	miRNA	Expression Level	Target genes	Functions	Clinical relevance	References
NK cell	miR-182	UP	NKG2D	↑NK killing activity	Hepatocellular Carcinoma	(15)
	miR-155	UP	INPP5D/SHIP1	↓Cell survival and Cell-cycle progression	Lymphoma	(16)
	miR-21	UP	PTEN	↓Cell survival	Lymphoma	(16)
	miR-342-3p	DOWN	Bcl-2	↑Cell apoptosis	Non-small Cell Lung Cancer	(17)
	miR-30b	DOWN	CCL22	↑Cell apoptosis	Lymphoma	(18)
	miR-218-5p	UP	SHMT1	↓IFN-γ and TNF-α expression ↓Cytotoxicity	Lung Adenocarcinoma	(19)
M1 Macrophage	miR-21	UP	STAT3	↓M2 polarization	Inflammation	(20)
			IFN-γ/ STAT1	↓PD-L1 expression ↓M1 polarization	Melanoma	(21)
	miR-342-5p	UP	Bmpr2, Akt1	↑Inflammatory stimulation	Atherosclerosis	(22)
			CXCL12	↓Recruitment of macrophages, ↓tumor angiogenesis	Tumor	(23)
M2 Macrophage	miR-195	UP	Notch2	↓M2 polarization	colorectal cancer	(24)
	miR-101	UP	C/EBPα, KLF6	↓M1 polarization ↑Proliferation and invasion	Breast Cancer Ovarian Cancer	(25)
DC	miR-5119	DOWN	PD-L1, IDO2	↓T cell exhaustion	Breast Cancer	(26)
	miR-155	UP	c-Fos	↓Proinflammatory cytokine production		(27)
	miR-564	UP	TP53	↑Proliferation and migration of DC	Systemic lUPus erythematosus	(28)
	miR-320	UP	EGR3	↓Invasion and metastasis	Glioblastoma	(29, 30)
			TWIST1	↑Cell proliferation and invasion	Ovarian Cancer	(29, 31)
T Cell naive	miR-150	UP	LKB1	↑Proliferation and migration; ↓Apoptosis	Non-small Cell Lung Cancer	(32, 33)
	miR-16	UP	ETS1	↑Proliferation, migration and invasion	Melanoma	(34, 35)
	miR-142-3p	UP	HMGA2	↑Apoptosis G2/M cell cycle arrest	Breast Cancer	(34, 36)
	let-7f	UP	Integrin β1	↓Viability, migration and invasion; ↑Apoptosis	Non-small Cell Lung Cancer	(34, 37)
T Cell exhausted	miR-155	UP	CTLA-4	↓T cell proliferation	Atopic Dermatitis	(38, 39)
	miR-26	DOWN	PD-1	↓T cells exhaustion	Melanoma	(39, 40)

### 2.1 NK Cells

The most distinguishing feature of NK cells is that their ability to kill without first recognizing tumor-specific antigens (41). Instead of reprogramming themselves to be less immunogenic, tumor cells could avoid clearance by altering the miRNA expression profile to inhibit the activation of NK cells. Additionally, because the balance between activation and inhibition of NK cell receptors determines whether NK cells are activated (42), miRNAs can contribute to tumor cells’ immune escape by affecting natural killer group 2 member D (NKG2D), one of the major activating receptors of NK cells, or its 8 ligands (NKG2DL). NKG2DL contains major histocompatibility complex (MHC) class I-chain related molecules A (MICA), B (MICB) and the UL16 binding proteins (ULBP)1-6 are included in NKG2DL.

The translation process of NKG2D and cytotoxicity of NK cells would be significantly reduced in the cell lines of over-expressing miR-1245 (43). Similarly, miRNAs can inhibit NKG2DL translation. Research has demonstrated higher miR-20a levels in ovarian tissues of ovarian cancer patients, than in the normal group. In an *in vitro* model, miR-20a targets 3’UTR of MICA/B, resulting in reduced activation signaling and decreased NK cells cytotoxic activity. In addition, miR-20a could also specifically inhibit MAPK1 (ERK2) which is upstream of ULBP2 in breast cancer cells, and could similarly impair the cytotoxicity of NK cells. In addition, miRNAs can also target ULBP2 to involve this progress. For instance, ULBP2 can be targeted by miR-34 as well as miR-449 family members to inhibit translation, thereby affecting NK cell function (44).

### 2.2 Macrophages

Under different conditions, macrophages, as immune cells with phagocytic functions *in vivo*, can polarize into two phenotypes with opposite functions. There are two types of macrophages: classically activated (M1) macrophages, which suppress tumors, and alternatively activated (M2) macrophages, which promote tumors (45). Because of this feature of macrophages, tumor cells can convert macrophages gathered in tumor tissues to the M2 phenotype by expressing specific miRNAs, resulting in the formation of immunosuppressive TME with a greater chance of immune escape.

#### 2.2.1 NF-κB

NF-κB is required for lipopolysaccharide (LPS) stimulated macrophage polarization toward M1 (46). miRNAs can regulate the direction of macrophage polarization by affecting transcription factors in the NF-κB pathway. D’ Adhemar et al. demonstrated thatmiR-21 and miR-146a can influence the regulatory role of myeloid differentiation factor 88 (MyD88) in the toll-like receptor 4 (TLR4) pathway in ovarian cancer by inhibiting MyD88 translation (47). As a result, miRNAs regulation of MyD88 affects downstream NF-κB activation, inhibiting the macrophage polarization toward the M1 phenotype. CYLD is a deubiquitinase that inhibits IKK activation by reducing TRAF2 and Nemo ubiquitination, similar to an NF-κB inhibitor. Meanwhile, miR-182 can target CYLD directly and inhibit its translation, activating NF-κB (48). The data show that miR-182 is one of the key regulators that promote M1 macrophage polarization.

#### 2.2.2 STATs

STATs, a class of transcription factors that can bind to the promoters of target genes, are grouped into 7 subtypes. Members of the STATs protein family play critical roles in transcription factors mediating M1/M2 polarization in macrophages. miR-21, a key regulator of apoptosis and tumor progression, can be overexpressed in tumor-stimulated macrophages and downregulate STAT1 and Janus kinase 2 (JAK2). Following a reduction in STAT1 and JAK2 levels, macrophages are unable to form JAK2-catalyzed dimerized STATs, as much, they enter the nucleus to bind to cis-acting elements in target gene promoter regions, inhibiting M1 polarization and greatly reducing antitumor capacity (21).

#### 2.2.3 PPARs

PPARs are a family of nuclear transcription factors, and macrophages can be induced to polarize toward M2 upon activation of PPAR-γ, an isodorm of PPARs. This is because PPAR-γ can directly binds to the p65/p50 subunit of NF-κB, forming a transcriptional repressor complex that inhibits the expression of NF-κB. miR-130a expression was found to be decreased in patients with non-small cell lung cancer, and the expression of miR-130a in M1 was higher than that in M2. Further findings showed that miR-130a inhibits macrophage polarization toward M2 and enhances M1 polarization by reducing PPAR-γ expression (49).

### 2.3 DCs

DCs are the most powerful antigen-presenting cells in the body, serving as a link between innate and adaptive immunity. However, in TME, dysregulated miRNAs frequently affect their maturation and function. In this case, DCs are frequently transformed into immune negative regulators, which aid tumors to escape immune surveillance (50).

It has been reported that miR-155 is a key gene in the maturation of breast cancer DCs. Since c-Fos and arginase-2, two transcription factors essential for DC maturation and function, were shown to be targets of miR-155. In addition, miR-155 also epigenetically regulates CCR7 in DCs, which is key to induce the migration of mature DCs to the T cell zone of draining lymph nodes (51). RT-PCR and deep sequencing show that miRNAs are indeed abnormally expressed in plasma and tissues of gastric cancer patients, with only miR-17-5p exerting an oncogenic effect in gastric cancer. Gastric cancer-derived miR-17-5p could be taken up by immature DCs, inhibiting LPS-induced DCs maturation and endocytosis activity, promoting gastric cancer development in two ways (52).

## 3 miRNA-Regulated Specific Immune Response *via* Affecting Naive T Cells

The specific immune response begins gradually, but it shows high specificity and plays an important role in the immune mechanism against tumors. The normal activation and proliferation of T cells, in particular, is a complex process of signal stimulation and transduction that is required for the body to clear tumor cells *via* specific immunity. miRNAs, as an important regulator involved in the majority of physiological activities of the body, are also key factors for T cell activation. miRNAs can participate in the signal transduction process of TCR and costimulatory molecules, separately or simultaneously, through various mechanisms that influence the activation process of naive T cells; this consequently, regulates the initiation of cellular immunity (**Table 1**). Correspondingly, when T cells are not sufficiently activated and differentiated to initiate cellular immunity, tumor cells can escape immune surveillance and rapidly proliferate and deteriorate.

### 3.1 T-Cell Receptor (TCR)

The first step toward full T cell activation is the binding of pMHC (a complex of antigenic peptide and MHC) to the TCR and its delivery to the cell. The TCR and its proximal signaling molecule can be considered as a “signal integrator”, receiving and integrating positive and negative signals induced by the stimulus and thus determining the role of TCR. Mounting evidence in recent years has shown that some stage-specific expression of miRNAs may act as fine-tuners for T cell development by influencing signal transduction downstream of the TCR (53). Positive stimulation is generally provided by the pMHC in conjunction with the TCR, whereas negative signaling is controlled in part by multiple phosphatases downstream of the TCR; this set an activation threshold for T cells where negative regulation may occupy a dominant position. A negative signal is inhibited when tumor- secreted miRNAs target these phosphatases, causing an increasing in TCR signal intensity.

Mounting evidence has shown a role for miR-20a in inhibiting transcriptional activation in T cell lines and that miR-20a is specifically upregulated during T cell activation. TCR stimulation was found to rapidly induce miR-20a expression. Following specific overexpression in some tumor tissues, miR-20a negatively regulates TCR signaling by inhibiting the phosphorylation of ZAP70, LAT, PLC-γ, and Erk as well as the expression of CD96. Additionally, miR-20a can regulate cellular immunity by inhibiting the expression of cytokines such as IL-10, IL-2, and IL-4 (54). However, the mechanism is still unknown, and it may be related to the fact that interleukin secretion is dependent on TCR-mediated ERK1/2 and Ca++ signaling pathways (55). Also, miR-181a can regulate TCR signaling intensity at the post-transcriptional level. miR-181a increases TCR signaling intensity by inhibiting several phosphatases, including dUSP5, dUSP6, SHP2 as well as PTPN2, that negatively regulate the TCR signal network (56).

### 3.2 Costimulatory Molecules

T cell costimulatory signals in addition the first signal provided by the binding of the TCR to pMHC, are required to fully activate naive T cells and initiate specific immunity (57). T cells will become an anergic state or even apoptotic if a costimulatory molecule is not present to provide the second signal. T cell surface receptors that bind to specific ligands on the APC provide costimulatory signals that include both activating (CD28) and inhibitory (PD-1 and CTLA-4) signals (58). Unfortunately, tumor cells frequently suppress the expression of co-stimulatory molecules on their surface by aberrantly expressing miRNAs, causing naive T cells to fail to fully activate and efficiently initiate specific immune responses, resulting immune escape (59). Costimulatory signal pathways primarily include the CD28/B7 pathway and CD40/CD40L pathway. Of note, the CD28/B7 pathway is one of the most distinctive costimulatory signaling pathways. CD28 binding to its ligand CD80/CD86, or CD40 binding to CD40L, can send critical second signals that fully activate naive T cells. CD40 binding to CD40L promotes the expression of CD80, CD86 on APCs, whereas CD28 binding to CD80/CD86 upregulates CD40L, resulting in a positive feedback effect that increases the activation efficiency of naive T cells (60).

Compelling evidence shows that miRNAs almost always regulate costimulatory molecules directly or indirectly in various pathological conditions (61). miR-134 acts as an immune escape facilitator in melanoma cells, not only directly targeting B7-2 to reduce its expression, but also mediating the reduction of interferon-γ (IFN-γ) and tumor necrosis factor-α (TNF-α) levels produced by lymphocytes (62). Also, miR-145 can target CD28, resulting in decreased CD28 expression.

## 4 miRNA-Regulated Apoptotic Process of Tumor Cells

**Figure 2:**
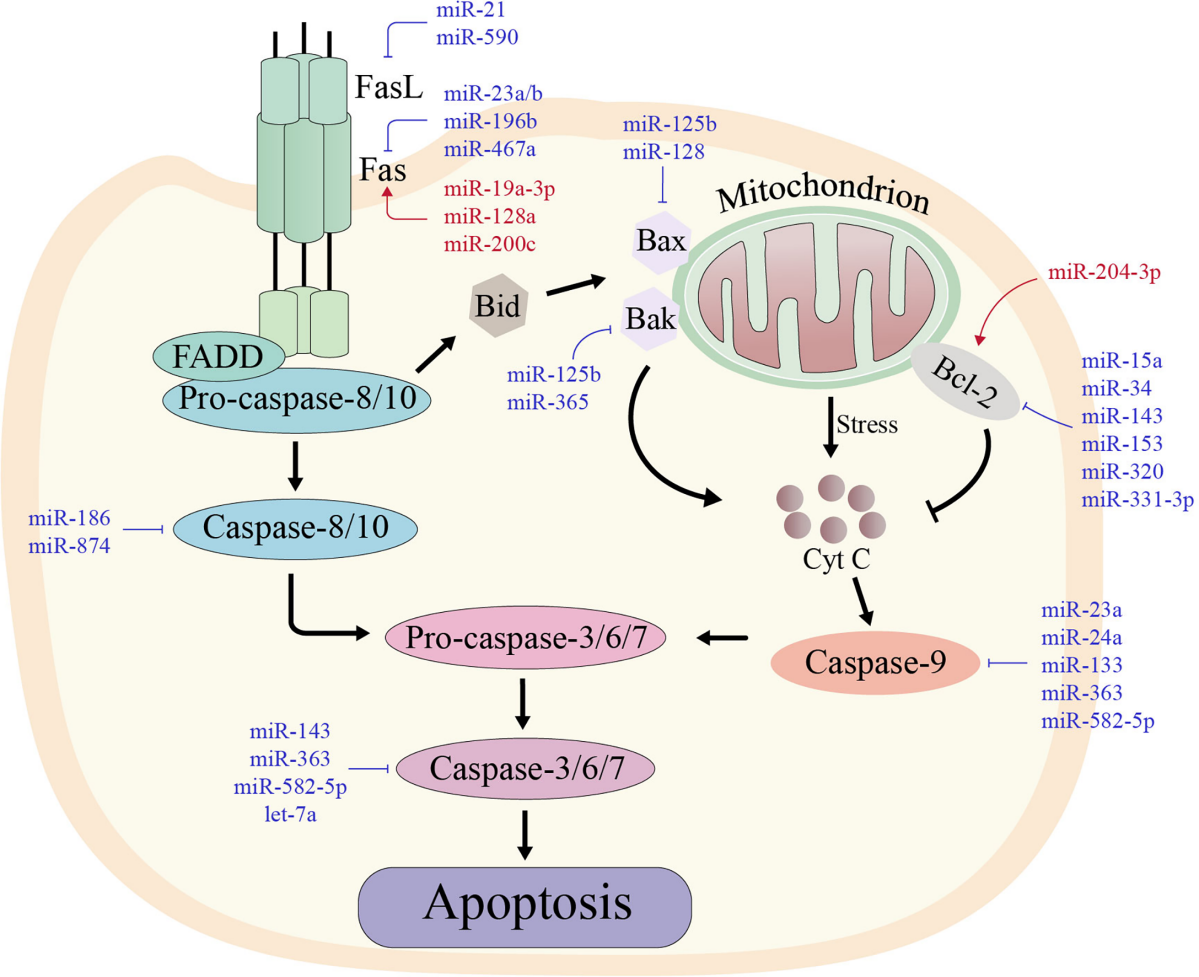
The two major apoptotic pathways with their regulating microRNAs in various types of cancers. The red miRNAs represent a promoting role, while the blue miRNAs play an inhibiting role. Left: In the extrinsic pathway of apoptosis, death-inducing factors such as FAS ligand (FASL) bind to its receptor (FAS) and recruit FAS-associated death structural protein (FADD) and pro-caspase-8. Cleavage and activation of pro-caspase-8 then activates the downstream caspase cascade, leading to sequence activation of caspases-3, -6 and -7. Right: In the intrinsic pathway of apoptosis, some stimuli disrupt the balance between pro-apoptotic proteins and anti-apoptotic proteins, which stimulates the release of cytochrome c in mitochondria. Cytochrome c forms an apoptotic complex with caspase-9, which is then cleaved by mature caspase-9 produced by the complex to form mature active caspase-3. Thus, both extrinsic and intrinsic apoptotic pathways lead to the activation of caspase-3, which cleaves more than 500 cytoplasmic proteins to induce apoptosis.

Apoptosis is an autonomous mode of death regulated by genes, and the ability of tumor cells to escape apoptosis is their distinguishing feature (63). Tumor cells can mediate immune escape by inhibiting various proteins involved in the apoptotic process *via* the inhibitory effects of miRNAs. miRNAs, for examples, can resist apoptosis by increasing the expression of anti-apoptotic molecules, including B-cell lymphoma-2 (Bcl-2), and inhibiting pro-apoptotic molecules such as death receptors. The mechanisms involved in the regulation of apoptosis by miRNAs from death receptors and mitochondrial apoptosis pathways will be reviewed below (**Figure 2**).

### 4.1 Death Receptors Pathways

Apoptosis mediated primarily by death receptors on the cell membrane is referred to as the death receptor pathwys. Death receptors are tumor necrosis factor superfamily cell surface markers, and Fas/Fas ligand (FasL) is a well-known combination. And tumor cells secrete miRNAs that can affect Fas or FasL, which results in a downstream cascade response caused by Fas and FasL binding that fails to activate effector caspases, such as Caspase-3, which inhibits apoptosis in tumor cells.

Fas has been found to be frequently downregulated during tumor development, causing tumor cell resistance to apoptosis. miRNAs that are abnormally expressed in most tumor tissues serve as important regulators involved in the downregulation of Fas. The expression of miR-23a, miR-23b, and miR-467a is upregulated in radiation-induced thymic lymphoma (64, 65). Fas is the direct target of miR-23a/b and miR-467a. Studies showed decreased levels of Fas following overexpression of miR-23a/b, inhibiting apoptosis; however, miR-23a inhibited Fas more strongly than miR-23b. Furthermore, in rectal cancer, both the lowly expressed miR-19a-3p, and the highly expressed miR-196b, can directly regulate expression by directly targeting Fas, thereby participating in the apoptotic process of tumor cells (66, 67). miRNAs, which are known for their ability to target Fas, can also target Caspases to participate in regulating the extrinsic apoptosis pathway. Overexpression of miR-582-5p and miR-363 in glioblastoma directly targets Caspase-3 and Caspase-9, impacting apoptosis (68).

### 4.2 Mitochondrial Apoptosis Pathway

The permeability of the outer membrane increases when mitochondria receive signals from intracellular stimuli, allowing the membrane gap protein component cytochrome C (cyt-C) to enter the cytoplasmic matrix; this triggers apoptosis. Mitochondrial membrane permeability is greatly regulated by miRNAs aberrantly secreted by tumor cells through the Bcl-2 protein family.

Bcl-2 is a key regulator of mitochondrial outer membrane permeability, which takes part in tumor cell apoptosis by regulating cyt-C release and Caspase activation. miRNAs abnormally overexpress Bcl-2 in multiple malignancies, including prostate cancer, osteosarcoma cancer, and breast cancer. For example, miR-204-5p expression is abnormally downregulated in various cancers, particularly prostate cancer. Lin et al. demonstrated that the direct target of miR-204-5p in prostate cancer was Bcl-2, and inhibiting Bcl-2 expression levels decreased tumor cell viability and induced apoptosis, demonstrating that miRNA could regulate apoptosis by regulating Bcl-2 expression levels (69). By directly targeting Bcl-2, miR-143 can downregulate its expression, which is aberrantly downregulated in osteosarcoma carcinoma. Meanwhile, miR-143 can activate Caspase-3, a downstream signal of Bcl-2, and is associated with increased expression of pro-apoptotic genes PARP, Bcl-2-associated X protein (Bax), Bcl-2 antagonist killer (Bak), and Bcl-2 cell death agonist (Bad), all of which induce apoptosis in tumor cells (70).

## 5 Potential Applications of miRNAs in Tumor Immune Escape

miRNA regulation has emerged as one of the central elements in the complicated multistep process of immune escape. Since tumors are typically caused by mutations in multiple genes, the ability of miRNAs to simultaneously target more than one gene involved in tumor development or escape is the primary advantage of miRNA-based therapeutic approaches. That is, one of the most attractive and specific strategies for their use as a therapeutic agent is their ability to achieve widespread silencing of pro-tumoral pathways with a small number of miRNAs (11). In addition, miRNAs are endogenous antisense nucleotides with lower toxicity and immunogenicity than protein-based drug complexes or plasmid DNA-based gene therapies. In this context, miRNA-based applications are being developed either alone or in conjunction with ongoing immunotherapies, which could significantly contribute to the success rate of future tumor treatments (11, 71).

### 5.1 Therapeutics Using miRNAs Alone

miRNAs function as tumor suppressors or oncomiRs, and their dysregulation can promote tumor development and escape. As a result, therapeutics based solely on miRNAs are classified into two approaches: (i) restoring the expression of tumor suppressor miRNAs *via* small molecules or miRNA mimics; (ii) blocking the function of oncomiRs *via* miRNA antagonists, such as antagomiRs, LNA, and miRNA sponge (72).

#### 5.1.1 Restoring Tumor Suppressor miRNA Levels

Restoring tumor suppressor miRNAs can be accomplished either indirectly through small molecule drugs, or directly by administering miRNA mimics to affect the molecular pathways of miRNA biogenesis (71).

Hypermethylation of CpG island promoters, which leads to transcriptional silencing of tumor suppressor genes, has become a hallmark phenomenon in cancer cells (73). Furthermore, because the same epigenetic interference is seen in tumor suppressor miRNAs, it is possible to reverse the epigenetic silencing of miRNAs using hypomethylating agents. Commonly used hypomethylating agents in the clinic such as, decitabine and azacitidine, can re-induce miRNAs expression (74). Furthermore, tumor suppressor miRNAs can be restored by enhancing miRNAs biogenesis. Enoxacin, a small-molecule antibacterial agent, has been shown to bind to the miRNA biosynthesis protein TARBP2 and promote the production of miRNAs (75).

miRNA mimics are more targeted than broad-spectrum miRNA restoring small molecules because they enhance the designated miRNAs. miRNAs mimics are double-stranded oligonucleotides of approximately 22-mer length, containing the same endogenous mature miRNA or its precursor sequence. Once transfected into cells, miRNAs are converted to a single-stranded form and participate in various physiological processes through miRNA-like functions (76). MRX34, a liposomal formulation of miR-34a, was the first miRNA therapy to enter the clinic and is mainly used in advanced or metastatic hepatocellular carcinoma. miR-34a acts as a tumor suppressor, regulating the downregulation of various genes involved in tumor immune escape. Evidence from preclinical studies showed that tumor growth was significantly inhibited in mice after tail vein injection of MRX34, with a great improvement in the survival rate (77). However, it was subsequently closed due to severe immune-mediated adverse events (78). Although the clinical trial was closed, the results of pharmacodynamics and efficacy might provide proof of concept for miRNA-based therapy.

#### 5.1.2 Blocking oncomiR Functions

The current strategy for inhibiting oncomiR function is primarily based on antisense miRNAs, which specifically suppress miRNAs that are aberrantly upregulated in tumor cells such as antagomiRs, LNA, miRNA sponges, etc. (71, 79, 80).

AntagomiRs are synthetic RNAs that have been chemically modified with 2’-O-methyl bonds and are coupled with cholesterol. AntagomiRs can function as anti-miRNAs that are completely complementary to the target miRNA sequence, preventing them from binding to target mRNAs (81). Although AMO and antagomiRs have similar anti-miRNA mechanisms, they differ in length and chemical modification, and both strategies have been studied preclinically in animal models (82).

LNAs have the highest affinity for target miRNAs due to the addition of methylene bridges connecting 2-O and 4-C atoms, leading to “locked” modifications of their ribose rings (71). A number of LNA-based methods are also undergoing preclinical studies. LNA-anti-miR-380-5p proved effective in reducing tumor size in an orthotopic mouse model of neuroblastoma (83). Miravirsen (also known as SPC3649), an LNA-based antisense molecule targeting miR-122, is undergoing clinical phase I and phase II trials in the treatment of hepatitis C (84, 85).

The miRNA sponge is a single mRNA with several tandem binding sites in its 3’UTR that is designed as a highly efficient molecule for long-term repression of miRNA genes. Due to the way miRNAs bind to their targets, a single type of sponge may shut down all miRNAs in the family with close affinity. miRNA sponges are already used to study miRNAs linked to tumor metastasis. Evidence shows that sponges targeting miR-9, miR-10b, and miR-31 can effectively reduce the expression of their respective target miRNAs, and have an ameliorative effect on breast cancer metastasis (86). However, because miRNA sponges are non-chemically modified competitive mRNAs, they have less affinity for target miRNAs than chemically modified LNAs and antagomiRs; as such, the required concentration may be increased. But whether excessive concentrations of miRNA sponges are associated with side effects is not known at this time (81).

### 5.2 miRNAs Are Employed as Adjuvants for Immunotherapy

Over the past few decades, the clinical application of immunotherapy has made tremendous progress and has become one of the most prosperous fields of cancer research and development. Several immunotherapeutic approaches, including adoptive cell therapy (ACT), immune checkpoints therapy, and cytokine therapy, have made significant clinical progress, particularly immune checkpoint therapy, with the successful marketing of Ipilimumab, a monoclonal antibody for treating melanoma and lung cancer. However, they have drawbacks such as side effects, ineffectiveness, and sometimes no effect on a significant proportion of patients (87, 88). Intriguingly, miRNAs, a widely used therapeutic candidate molecule, can act as an adjuvant to several major immunotherapies, synergistically increasing their efficacy potential while reducing their dosage. Here, we highlight the mechanisms of miRNAs as adjuvants in the ACT and immune checkpoint therapy, two of the most widely studied and clinically applied immunotherapies today.

#### 5.2.1 ACT

ACT is a type of immunotherapy in which autogenous immune cells are proliferated and/or modified outside the body before being infused back into the body. Adoptive T cell-based immunotherapy has proven to be effective in treating solid cancers and advanced hematologic malignancies (89). However, for a large group of patients, this approach fails to produce long-term effects, whereas in some cases, there is no effect at all, and the overall response rate is not satisfactory. The effectiveness of ACT may be enhanced by utilizing miRNAs to improve both T cell fitness and effector capacity (90).

“T cell adaptability”, referring to the ability of T cells to survive after transfusion, is an important factor influencing the effectiveness of ACT, and the fitness of adoptive T cells is dependent on their ability in response to regulatory factors. To enhance the fitness of T cells, miRNAs can be combined with ACT, and one approach is to antagonize miRNAs that act as negative regulators of T cell immune responses. Overexpression of miR-155 in lymphocytes has been demonstrated to improve T cell responsiveness to homologous γc cytokines, resulting in improved T cell survival and antitumor effects. miR-155 promotes cytokine production and signaling transduction through targeting several negative regulators upstream of the PI3K/AKT and STAT pathways (91). Antagonize miRNAs, which are negative regulators of T cell immune responses, is another way to improve T cell state. miR-146a is upregulated when T cells are activated and prevents the action of T cell overactivation by targeting TRAF6 and IRAK1 of the NF-κB signaling pathway and inhibiting TCR-induced NF-κB activity (92). Therefore, miRNA therapies such as antagomiRs and miRNA sponges that target miR-146a can then be used to enhance NF-κB activity in the adoptive T cells; this improves their fitness and may increased the response rate of ACT.

Another possible explanation for the low ACT responsiveness is that the adoptive T cells are exposed to an immunosuppressive environment in the tumor microenvironment, preventing them from releasing large quantities of pro-inflammatory cytokines and exerting powerful cytotoxic effects (93). If the levels of the specified miRNAs were artificially adjusted to increase the cytotoxic effect of the adoptive T cells, the response rate and persistence of ACT should theoretically be improved. The level of miR-23a was negatively correlated with the cytotoxic chemokines such as granzyme B and IFN-γ in cytotoxic T lymphocytes (CTL). The response rate and survival rate of melanoma mice in the miR-23a overexpression group were significantly lower than in the ACT group, possibly due to the ability of miR-23a to inhibit the expression of granzyme B and T-bet in CTL (94). Hence, inhibitory therapeutics targeting miR-23a can be used synergistically with ACT, enhancing the cytotoxic effects of adoptive T cells and improving ACT response rate and persistence. Overall, there is some evidence that miRNAs-based therapies have the potential as effective adjuncts to ACT, but their efficacy and whether they have adverse effects remain to be seen.

#### 5.2.2 Immune Checkpoint Therapy

Immune checkpoints are molecules that can play a suppressive role in the immune system, and tumors have been shown to express some immune checkpoints as triggers for immune escape. Given that the mechanism of most immune checkpoints is ligand-receptor interaction, they are easily blocked by antibodies or affected by other factors, thereby resulting in inability to function, which is immune checkpoint therapy (95). It is worth noting that an immune checkpoint inhibitor is a monoclonal antibody targeting immune checkpoints. Since the approval of Ipilimumab, an anti-CTLA-4 monoclonal antibody, for the treatment of metastatic and unresectable melanoma in 2011, seven immune checkpoint inhibitors have been recommended for the clinic and thousands of clinical trials are underway, which makes immune checkpoint therapy to be one of the most effective and cutting-edge approaches of cancer treatment. However, its clinical application is hampered by side effects, new immunotoxicity, and non-persistence of immune checkpoint. There is growing evidence indicating that miRNAs and immune checkpoint molecules are closely related, thus, combining them into one therapy has the potential to improve their efficacy.

PD-L1 on CD8T cells in diffuse large B-cell lymphoma can be induced by MALAT1, a miR-195 inhibitor. The proliferation as well as cytotoxic effects of CD8T cells with high PD-L1 expression were attenuated, suggesting that the combination of miR-195 mimics and PD-L1 inhibitors might be more effective in anti-cancer (96). In addition, inhibition of miR-28 led to a concomitant increase in three immune checkpoint receptors (PD-1, TIM-3 and LAG3) and induced a decrease in T cell secretion of IL-2 and TNF-α, reflecting the regulatory role of miR-28 in T cell depletion (97). Recently, a study showed that the combined application of miR-200c and BRAF inhibitors that were delivered to the designated sites by nanotargeted vehicles could effectively downregulate PD-L1 expression and increase the resistance of tumor cells to immune checkpoint inhibitors (98). However, although miRNAs are becoming popular in the field of immunotherapy as adjuvants for immune checkpoint therapy, further studies should be conducted to elucidate the mechanisms underlying the association between miRNAs and immune checkpoints.

## 6 Vehicles for miRNA-Based Therapeutic Strategies

**Figure 3:**
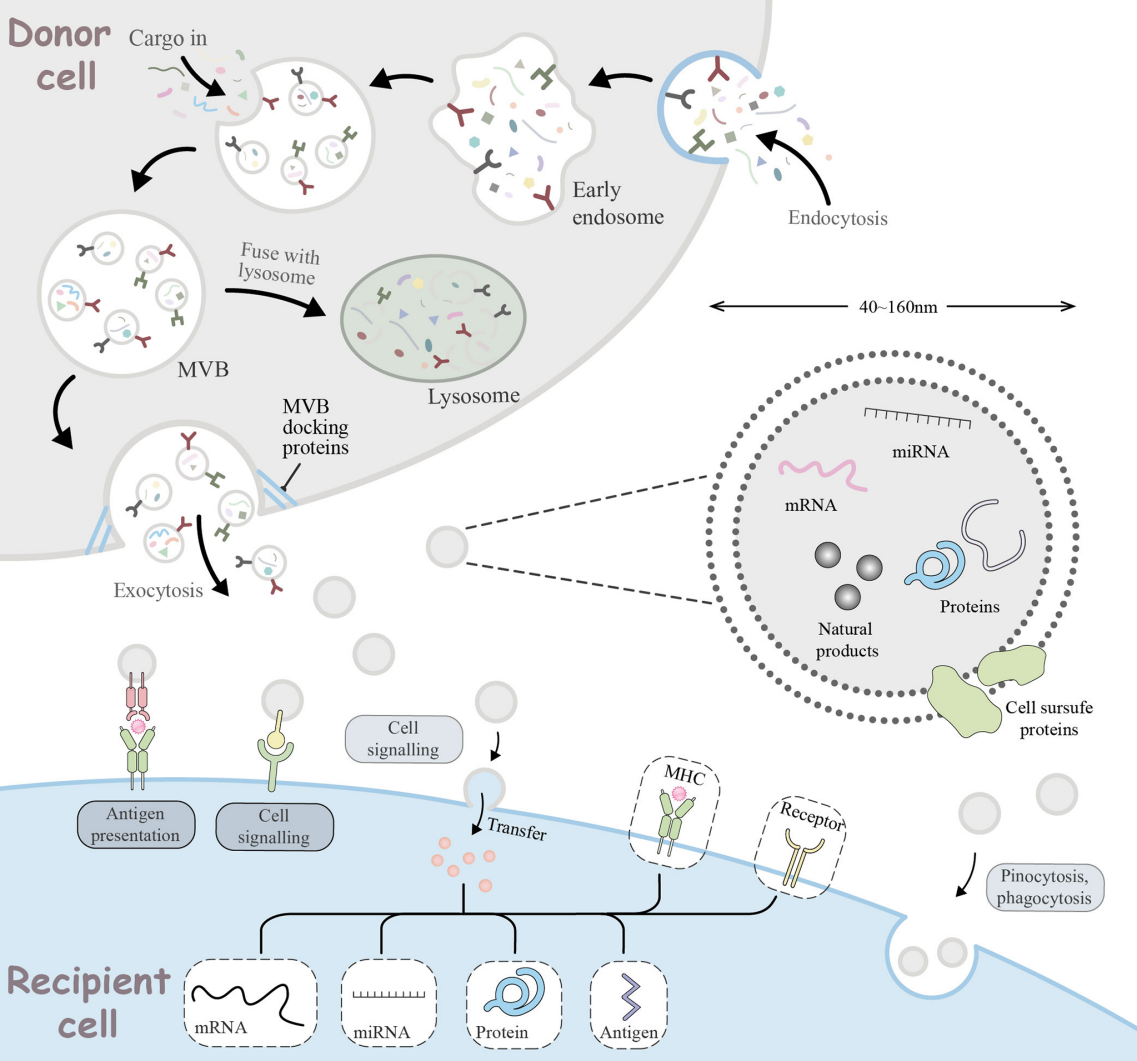
Cellular journey of exosomes from donor cells to recipient cells. The range of basic mechanisms of exosome biogenesis and their multiple ways of interaction with recipient cells. Extracellular components, such as proteins, lipids, metabolites, small molecules and ions, can enter the cell together with cell surface receptors *via* endocytosis to form early endosomes (EE). During EE maturation, the contents of the cell cytoplasm are sorted and loaded into vesicles, while the membrane of the EE invaginates, forming multivesicular bodies (MVB). This step can lead to further modification of the cargo, with cytoplasmic components entering the newly formed MVB. MVB can be degraded by lysosomes or transported to the plasma membrane *via* the cytoskeleton and microtubule network. With the help of MVB docking proteins, they are released through exocytosis which are referred to as exosomes. Exosomes range in size from 40 to 160 nm and usually contain RNA, miRNA, natural products, proteins, and cell surface proteins. Released exosomes can be seen as signal bodies involved in a variety of biological processes. They can be involved in antigen presentation processes, intercellular signaling, transfer or pinocytosis, phagocytosis into the recipient cell to deliver effectors for biological functions.

Insights into the role of miRNAs in tumor immune escape have led to their emergence as attractive novel therapeutic tools. However, miRNA-based therapies like miRNA mimics and antagomiRs are unstable *in vivo* due to the fact that naked miRNAs are rapidly degraded by nucleases. Moreover, their hydrophilicity, negative charge, and large physical size limits their ability to passively diffuse into the tumor cell, leading to unfavorable pharmacokinetics of miRNA-based therapy. Moreover, considering the way miRNAs binding to target genes, they may cause strong toxicity to normal cells if they are not targeted to specific sites (81). Therefore, miRNA-based therapy requires a safe, effective, and targeted drug vehicle to protect them from degradation and facilitate their targeted delivery *in vivo*, thus inducing desired gene regulation. To this end, various traditional vehicles such as liposomes, nanoparticles, and viruses have been used, but all have revealed some limitations in their biological properties and safety issues that have put them into doubt. The recent introduction of exosomes as vehicles may provide a new opportunity for miRNA-based therapy. As endogenous substances, exosomes have a good stability and biocompability of the internal environment and an intrinsic ability to cross physical barriers. Moreover, during biogenesis, exosomes can package nucleic acids as well as proteins, and internalize donor cell receptors on the surface and express them on the membrane, acting as shuttle carriers for intercellular communication *in vivo*. All these characteristics provide strong support for exosomes in defeating traditional vectors to provide new opportunities for miRNA-based therapy (**Figure 3**).

### 6.1 Current Commonly Used Vehicles for miRNAs Delivery

Liposomes are the most widely used and studied drug vehicles. For instance, MRX34, which was the first miRNA mimic to enter clinical trials, uses liposomes as a vehicle. It should be noted that a liposome is a tiny vesicle that can encapsulate the drug within a lipid-like bilayer. Although using liposomes as vehicles for drug delivery can improve drug stability and pharmacokinetics (99), they still have some disadvantages. For example, liposomes tend to accumulate in the liver and kidneys, and have to be removed by the mononuclear phagocytic system in a process that affects the phagocytosis of macrophages at higher doses. In addition, liposomes have been associated with acute hypersensitivity reactions in clinical use.

The use of viruses as vehicles is a relatively old strategy that possesses target specificity, efficient transgene expression, and the ability to cross biological barriers. The commonly used viruses include recombinant adeno-associated viruses (RAAV), adenoviruses, retroviruses and lentiviruses, which have achieved significantly results. For example, RAAV loaded with miR-134 achieved almost 100% transduction efficacy (100), and lentivirus expressing miR-7610 injected into tumors has been shown that it can suppress the growth of non-small cell lung cancer cells (101). However, the use of viruses as vectors has many drawbacks, particularly the immunogenicity of viruses and safety issues that can trigger oncogenic transformation, as well as the efficiency of gene transfer. A previous study showed with the use of lentiviruses as carriers may affect the changes in miRNA expression due to the integration sites of both the vector itself and the host genome (102). Besides, the availability of high-quality viral vectors in large quantities is a major obstacle to the large-scale application of viral vectors in clinic.

In recent years, inorganic nanoparticles have emerged as better drug vehicles compared to traditional liposomes and viral vectors because they have more advantages, such as adjustable size, ease of crossing biological barriers, more superior pharmacokinetics, and higher targeting. Zang et al. (103) invented a miRNA delivery system based on lipid-encapsulated calcium phosphate nanoparticles that can activate TAM and reverse its immunosuppressive phenotype to inhibit tumor progression. However, polymer coatings that confer superior pharmacokinetics and biodistribution often raise safety concerns, which limits their clinical translation (104).

### 6.2 Exosomes as a Promising Vehicle for miRNAs Delivery

Recent studies have revealed that membrane-based vesicular exosomes can transport various active biomolecules, including miRNAs, lipids, proteins and others, from donor cells to recipient cells under physiological and pathological conditions, ultimately altering the physiology of the recipient cell (13, 105, 106).

Exosomes, a kind of extracellular vesicles that range in size from 40 to 160 nm, are secreted by various cells including macrophages, dendritic cells, T cells, and B cells. One study reported that these cells can form early endosomes (EE) through invagination of the endosomal membrane (107). Over time, EEs form multivesicular bodies (MVBs) by invagination of the vesicle membrane, leading to the formation of multiple smaller vesicles and selective inward loading of nucleic acids, proteins and lipids into the cytoplasm. Generally, MVBs have two intracellular endings: (i) digestion by lysosomes; and (ii) fusion with the cell membrane and release into the extracellular matrix as exosomes. Notably, recipient cells can receive exosomes through ligand-receptor interaction, pinocytosis or membrane fusion (108).

Given that exosomes express protein molecules or lipid ligands on their surface, as well as molecules containing their own nucleic acids, proteins and lipids, they can shuttle through the body and serve a regulatory role in the physiological and pathological processes of various systems. For example, the presence of a plasma extracellular vesicle of non-tumor origin in melanoma patients may inhibit tumor cell proliferation through downregulation of beta-catenin and miR-34a delivery (109). The study also confirmed that endogenous exosomes can play a role in a number of diseases *in vivo* through their cargo loading capabilities and proteins on their membrane surface. In addition to endogenous exosomes being biomarkers of disease and prognostic factors, they have the potential to be carriers for delivering genes and drugs in clinical applications, particularly miRNA therapies. This can be attributed to the fact that their surface, unlike liposomes, has a complex and specific phospholipid bilayer structure, which enables them the property of loading both hydrophilic and lipophilic materials (110), allowing for more effective protection, longer circulation times, easier crossing of biological barriers (99, 111) and, most importantly, easier fusion with receptor cells to release drugs into them. Moreover, the protein molecules or lipid ligands expressed on their surface can help them to have a targeted function (112). Another highly appreciated feature of exosomes is their stability in circulation, as they have a negative charge on their surface and can avoid clearance from the mononuclear phagocytic system by expressing CD47 on their surface (113). In addition, the size of exosomes can be utilized for enhanced permeability and retention effect, making them more accessible to tumor tissues and long-term retention (114, 115). Collectively, these factors suggest that exosomes have great potential to overcome the shortcomings of the commonly used drug delivery systems and become the most effective vehicles of miRNAs therapeutics in the treatment of tumor immune escape.

Utilization of exosomes as vehicles of miRNAs largely depends on identification of an effective cargo loading strategy. It is well known that producing sufficient numbers of exosomes loaded with miRNAs has been a technical challenge (116). Currently, there are two main approaches that can be used to load cargo into exosomes: exogenous loading and endogenous loading (117). Exogenous loading, including electroporation, sonication, simple incubation, extrusion and freeze-thawing, and has been widely studied and practiced. However, exogenous loading techniques may cause exosome or cargo aggregation and may even change their morphological characteristics (118). This has resulted in endogenous loading gradually gaining acceptance. Although the process of loading exosomes under physiological conditions is not a completely random process, the exact mechanism of exosome sorting of miRNAs is still unclear (119). Based on this, the mechanism of exosomes sorting miRNAs can be used to load miRNAs into vesicles, thus achieving endogenous loading. Transfection is one of endogenous loading methods, involves encapsulating a gene in a vesicle during exosome biogenesis by overexpressing it in a donor cell (120). For example, miRNA or siRNA plasmids are loaded into exosomes by first transfecting with HEK293FT, and then isolating the exosomes (121). Exosomes carrying miR-1, miR-210, miR-214-3p and miR-21-5p, respectively, can exert the expected therapeutic effects by combining the miRNAs therein with target cells (122–125). In addition, several studies have demonstrated that RNA-binding proteins bind to particular miRNAs to enhance their loading in exosomes. For instance, the synaptophysin-binding cytoplasmic RNA-interacting protein (SYNCRIP) in hepatocytes and the sumoylated heterogeneous nuclear ribonucleoprote (hnRNPA2B1) in lymphocytes recognize specific GGCU and GGAG in miRNAs, respectively sequences (119). Consequently, the miRNAs with these sequences can increase the exosomes loading efficiency by binding to these two RNA binding proteins.

Although exosomes have made rapid and very promising progress in cell communication, clinical application of exosomes into clinical therapy as carriers of miRNAs is limited by several challenges. First, considering the specificity of miRNAs targeting, precise determination of the content of exosomes loaded with miRNAs should be to avoid side effects. Second, the mechanisms such as exosomes crossing the biological barrier and sorting miRNAs are not fully understood. Currently, the purification, loading, and storage technologies of exosomes are not very mature, and the cost effectiveness and yield are too small to be used in large-scale clinical therapies. However, it is evident that, with the progress of research, exosome-loaded miRNAs are likely to be a promising new strategy to address tumor immune escape.

## 7 Conclusion

Tumor immune escape is a key step in the malignant progression of tumors and is a significant factor for the failure of some tumor treatments. This review mainly focused on the effects of miRNAs interference on three aspects: innate immune response, specific immune response and apoptotic process. The paper has shown that miRNAs play a crucial role as oncogenic molecules and also tumor suppressor molecules in the tumor immune escape process. Currently, more and more nucleic acid drugs have entered the clinical research stage, thus, the superiority of miRNAs and their role in tumor immune escape should be exploited, alone or as adjuvants, for some developmental value. However, due to the instability of miRNAs in body fluid and demand for targeted delivery, diverse biomaterials as liposomes, viruses, inorganic nanoparticles and otherwise are applied as vehicles for miRNAs-based therapeutic drugs delivering. Especially, exosomes, an endogenous substance, can be equipped with nucleic acids and proteins for intercellular communication in both physiological or pathological conditions.

With the advancement of miRNA research, more and more studies are exploring the use of miRNA for diagnosis and prediction of prognosis, including miRNA-based therapies that use the expression patterns of some specific miRNAs to predict and target immune escape. The emergence of miRNAs can also be used to elucidate the mechanisms underlying tumor immune escape, with the overarching goal of understanding the malignant biological behavior of tumors.

Despite the great achievements in miRNAs-based therapeutic strategies, the structural properties of miRNAs and how they bind to target genes can cause many side effects that are inconsistent with the therapeutic goals. Therefore, in addition to chemical modification of miRNAs, identifying a biocompatible vehicle that can deliver miRNAs safely and efficiently to the specified location is a valuable solution to reduce side effects and off-targets. Accumulating evidence has suggested that, exosomes are very promising as vehicles of miRNAs. However, there are plenty of obvious issues that need further confirmation, such as the source of exosomes, isolation techniques, loading techniques, and what kind of drugs are suitable for loading. In conclusion, the era of small molecule RNA drugs is rapidly approaching, and we believe that, as research continues, miRNA-based drugs or therapies will be increasingly used in clinical treatment against tumors.

## Author Contributions

ZZ, YYL, CL, and TZ conceived the review. LY, QH, DZ, and YL were involved in the discussion. ZX, ZH, XL, and ZS contributed to the literature search. ZZ and YYL wrote the manuscript. All authors read and approved the final manuscript.

## Funding

This work was supported by the Beijing Natural Science Foundation [7202111], the National Science and Technology Major Project [2018ZX10101001-005-003] and Innovation Team and Talents Cultivation Program of National Administration of Traditional Chinese Medicine (No: ZYYCXTD-D-202005).

## Conflict of Interest

The authors declare that the research was conducted in the absence of any commercial or financial relationships that could be construed as a potential conflict of interest.

## Publisher’s Note

All claims expressed in this article are solely those of the authors and do not necessarily represent those of their affiliated organizations, or those of the publisher, the editors and the reviewers. Any product that may be evaluated in this article, or claim that may be made by its manufacturer, is not guaranteed or endorsed by the publisher.
